# Aiphanol, a native compound, suppresses angiogenesis via dual-targeting VEGFR2 and COX2

**DOI:** 10.1038/s41392-021-00739-5

**Published:** 2021-12-03

**Authors:** Shanmei Chen, Junnan Feng, Chuanke Zhao, Lixin Wang, Lin Meng, Caiyun Liu, Shaoqing Cai, Yanxing Jia, Like Qu, Chengchao Shou

**Affiliations:** 1 Key Laboratory of Carcinogenesis and Translational Research (Ministry of Education/Beijing), Department of Biochemistry and Molecular Biology, Peking University Cancer Hospital & Institute, Beijing, China; 2 Key Laboratory of Natural and Biomimetic Drugs, School of Pharmaceutical Sciences, Peking University, Beijing, China; 3 Present Address: Key Laboratory of Molecular Pathology, The Affiliated Cancer Hospital of Zhengzhou University, Zhengzhou, China

**Keywords:** Tumour angiogenesis, Target identification

**Dear Editor**,

Pathological neo-vascularization is a hallmark of cancer and several diseases. Accumulating evidence supports the notion that antiangiogenic treatment can abolish tumor angiogenesis to achieve longer disease-free survival. Although growth factors and their receptors function as the main drivers in angiogenesis, the involvement of other regulators, e.g., Cyclooxygenase-2 (COX2),^1^ should also be considered, especially for managing the resistance to therapies against receptor tyrosine kinases (RTKs). Hence, utilizing distinct inhibitors and developing multitargeting agents could be desired and practical approaches in conquering tumor angiogenesis.

Plants are rich in compounds with diverse biological functions. The anti-tumor and anti-inflammatory potentials of *Sarsaparilla*, aka *Smilax Glabra Rhizome* (SGR), were extensively studied,^2^ but its influence on angiogenesis has not been explored. Herein we found that SGR inhibited proliferation and motility of primary human umbilical vein endothelial cells (HUVECs) (Supplementary Fig. S1a–c). Importantly, SGR inhibited both basal and growth factors-stimulated angiogenesis in tube formation assay (Supplementary Fig. S1d), and this effect was dose-dependent (Supplementary Fig. S1e). We noticed that SGR contains some compounds with antiangiogenic capabilities.^2^ Aiphanol, originally separated from the seeds of *Aiphanes aculeate*,^3^ was also identified in SGR.^2^ Aiphanol represents an unprecedented scaffold of stilbenolignan in which one stilbene unit is connected with one phenylpropane moiety by a 1,4-dioxane bridge (Fig. 1a). To date, the biological effects of stilbenolignans, including Aiphanol, are largely unclear. Aiphanol was reported to inhibit angiogenesis in the rat aortic ring assay;^4^ however, the mechanism and its role in regulating tumor angiogenesis remain to be determined.

**Fig. 1:**
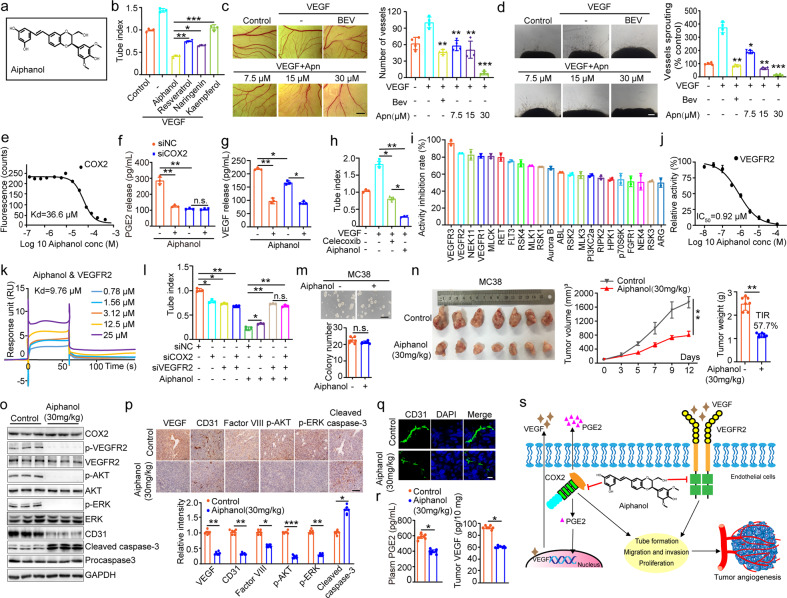
Aiphanol inhibits angiogenesis and tumor growth via dual-targeting VEGFR2 and COX2. **a** Structure of Aiphanol. **b** Effects of Aiphanol, Resveratrol, Narigenin, and Kaempferol (all at 30 µM) on VEGF-induced tube formation of HUVECs (*n* = 3 per group). The mesh numbers of tubular network were quantified and compared. **c** Aiphanol inhibited VEGF-induced formation of new vessels in the chicken embryo chorioallantoic membrane (CAM) assay (*n* = 4 per group). The numbers of vessels were counted and compared. VEGF-targeting antibody Bevacizumab (Bev, 0.5 mg/mL) was used as the positive control. Scale bar, 2 mm. **d** Aiphanol inhibited microvessels outgrowth in the mouse aortic rings assay (*n* = 4 per group). Microvessels’ area was calculated and compared. Bevacizumab (Bev, 0.5 mg/mL) was used as the positive control. Scale bar, 200 μm. **e** Microscale thermophoresis (MST) analysis of the binding affinity between Aiphanol and COX2. Kd was calculated by the curve fitting. **f, g** ELISA analysis of PGE2 (**f**) and VEGF (**g**) secreted by Aiphanol-treated HUVECs with or without COX2 sliencing (*n* = 3 per group). **h** Effects of Aiphanol and Celecoxib (both at 30 µM) on the tube formation of HUVECs (*n* = 3 per group). **i** List of kinases with >50% inhibition of activity by Aiphanol in the in vitro kinase profiler^TM^ assay. **j** The IC_50_ measurement of Aiphanol-inhibited VEGFR2 kinase in ADP-Glo^TM^ assay. **k** Surface plasma resonance (SPR) analysis of Aiphanol-VEGFR2 binding. **l** Tube formation analysis of HUVECs treated with Aiphanol following transfection with COX2 siRNA or/and VEGFR2 siRNA. Cells were transfected with indicated siRNA for 36 h, followed by tube formation assay for 6 h with or without treatment of 30 μM Aiphanol (*n* = 3 per group). **m** Soft-agar colony formation assay of Aiphanol-treated MC38 cells (*n* = 6 per group). Scale bar, 400 µm. **n** Single-dose (30 mg/kg) treatment of Aiphanol inhibited MC38 tumor growth in the syngeneic mouse model (*n* = 7 per group). Left, macroscopic photo of dissected tumors; middle, growth curves of tumors; right, comparison of tumor weight at the endpoint. TIR, tumor inhibition rate. **o** Western blot analysis of indicated proteins in MC38 tumor tissues after single-dose (30 mg/kg) treatment of Aiphanol (*n* = 3 per group). **p** IHC staining of indicated proteins in MC38 tumor tissues after single-dose (30 mg/kg) treatment of Aiphanol. Upper panel, representative staining for each protein, scale bar, 100 μm; lower panel, comparison of the intensity of indicated protein (*n* = 6 per group). **q** Immunofluorescent analysis of CD31 (green) in the frozen sections of MC38 tumor tissues after single-dose (30 mg/kg) treatment of Aiphanol. Nuclei were counterstained with DAPI (blue). Scale bar, 10 µm. **r** ELISA analysis of the levels of plasma PGE2 (left) and tumor VEGF (right) from mice treated with single-dose (30 mg/kg) of Aiphanol (*n* = 7 per group). **s** The schematic representation of the mechanism underlying Aiphanol-inhibited tumor angiogenesis via dual blocking VEGFR2 and COX2. Data are expressed as mean ± SEM. Statistical analyses used unpaired *t*-test or one-way ANOVA test. *P < 0.05; **P < 0.01; ***P < 0.001; n.s. no significance

We observed efficient uptake of Aiphanol by HUVECs (Supplementary Fig. S2a). Aiphanol inhibited growth factors, especially vascular endothelial growth factor (VEGF), induced tube formation of HUVECs (Supplementary Fig. S2b), which was dose-dependent (Supplementary Fig. S2c). Compared with other compounds from SGR with known antiangiogenic capacity,^2^ i.e., Resveratrol, Naringenin, and Kaempferol, Aiphanol exhibited stronger activity in preventing VEGF-induced tube formation (Fig. 1b and Supplementary Fig. S2d). Aiphanol also inhibited VEGF-induced tube formation of human microvascular endothelial cells (HMEC-1) and porcine aortic endothelial cells (PAEC) (Supplementary Fig. S2e). Moreover, Aiphanol inhibited proliferation of HUVECs (Supplementary Fig. S3a). Aiphanol had minimal impact on the cell-cycle profiles of HUVECs, but it induced apoptosis (Supplementary Fig. S3b–d) and decreased motility (Supplementary Fig. S3e, S3f). Furthermore, results of four angiogenesis models, including the chicken embryo chorioallantoic membrane assay (Fig. 1c), the mouse aortic ring assay (Fig. 1d), the vascular fluorescent transgenic zebrafish model (Supplementary Fig. S4a), and the Matrigel plug assay (Supplementary Fig. S4b), substantiated the antiangiogenic ability of Aiphanol in the ex vivo and in vivo systems.

Aiphanol could inhibit COX1/2 activities,^4^ but the mechanism and the biological significance are elusive. We verified Aiphanol-antagonized COX2 activity (IC_50_ = 2.7 μM) (Supplementary Fig. S5a). Microscale thermophoresis measurements demonstrated a direct Aiphanol-COX2 binding with the dissociation constant (Kd) of 36.6 μM (Fig. 1e). Structural simulation revealed that Aiphanol binds to the catalytic domain of COX2 (docking score = −8.118). Driven by the phenylpropane unit and the dioxane bridge, Aiphanol might inhibit COX2 activity through occupying its substrate binding pocket (Supplementary Fig. S5b, S5c). In Aiphanol-treated HUVECs, protein levels of COX2 remained unaffected (Supplementary Fig. S5d), but COX2’s enzymatic product, the inflammatory mediator Prostaglandin E2 (PGE2), and the downstream factor of PGE2-initiated signaling, VEGF, were reduced in the conditioned medium. After COX2 knockdown (Supplementary Fig. S5e), the inhibitory effects of Aiphanol on PGE2 and VEGF levels were markedly counteracted (Fig. 1f, g), validating Aiphanol-inhibited COX2 activity in cells. Compared with Celecoxib, a selective COX2 inhibitor, Aiphanol displayed stronger activity in blocking tube formation (Fig. 1h).

The stilbenes could repress kinase activities due to their structural characteristics.^5^ We hypothesized that Aiphanol may also inhibit kinase activity through its stilbene unit. Results of cell-ELISA showed that global phospho-serine/threonine and phospho-tyrosine signals were transiently diminished in Aiphanol-treated HUVECs (Supplementary Fig. S6a). By screening 201 diseases-related kinases, we found that Aiphanol strongly inhibited the activities of lymphangiogenesis-related kinase VEGFR3/FLT4, angiogenesis-related kinases VEGFR2/KDR and VEGFR1/FLT1. Meanwhile it weakly to moderately inhibited several other RTKS and members of PI3K-AKT and MAPK pathways (Fig. 1i and Supplementary Table S1). Because VEGFR1 depletion failed to override Aiphanol-inhibited tube formation, viability, or motility (Supplementary Fig. S6b–e), we then focused on the roles of VEGFR2. We validated Aiphanol-imposed inhibition on VEGFR2 kinase activity (IC_50_ = 0.92 μM) (Fig. 1j) and showed a direct VEGFR2-Aiphanol interaction (Kd = 9.76 μM) (Fig. 1k). Structural simulation predicted a docking score of −10.576 for this complex. Contributed by the stilbene and the phenylpropane unit, Aiphanol might target the VEGFR2’s ATP-binding domain, thereby preventing its activation (Supplementary Fig. S6f, S6g). Unlike VEGF-targeting antibody Bevacizumab, Aiphanol did not prevent VEGF-VEGFR2 interaction (Supplementary Fig. S6h). Nevertheless, Aiphanol treatment in HUVECs reduced VEGF-induced phosphorylations of VEGFR2, AKT, and ERK in a time- and dose-dependent manner (Supplementary Fig. S6i, S6j).

COX2 silencing partially reversed the inhibitory effect of Aiphanol on tube formation, but failed to ameliorate Aiphanol-inhibited viability or motility. However, VEGFR2 depletion significantly antagonized Aiphanol-inhibited tube formation, viability, and motility, and the effects of dual-silencing against VEGFR2 plus COX2 were similar to those of VEGFR2 knockdown (Fig. 1l and Supplementary Fig. S7a–d). Aiphanol-induced apoptosis of HUVECs was associated with upregulation of P53 and BAX (Supplementary Fig. S3d), which was weakened by VEGFR2 silencing (Supplementary Fig. S7e, S7f).

Next, we examined Aiphanol’s effect on tumor angiogenesis. Unlike HUVECs, MC38 murine colon cancer cells were deficient in VEGFR2 expression and insensitive to Aiphanol-induced caspase3 cleavage (Supplementary Fig. S8a). Besides, Aiphanol did not affect soft-agar colony/plate colony formation (Fig. 1m and Supplementary Fig. S8b), likely due to low Aiphanol uptake by MC38 cells (Supplementary Fig. S8c). However, MC38 tumor growth was retarded by oral administration of Aiphanol in the syngeneic mouse model (Fig. 1n and Supplementary Fig. S8d), correlating with enhanced apoptosis and decreased phosphorylations of VEGFR2, AKT, and ERK in tumor tissues (Fig. 1o, p, and Supplementary Fig. S8e). MC38 cells had no potential of vasculogenic mimicry (Supplementary Fig. S8f), but the levels of vascular markers, CD31 and Factor VIII, were reduced by Aiphanol (Fig. 1o–q and Supplementary Fig. S8e, S8g). We then concluded that Aiphanol’s inhibition on MC38 tumor growth was resulted from diminished angiogenesis. Additionally, PGE2 levels in the plasma and VEGF levels in tumor tissues were lowered by Aiphanol (Fig. 1r), signifying that COX2 activity was inhibited in vivo. Meanwhile, no significant changes in body weight or the morphologies of major organs of Aiphanol-treated mice were detected (Supplementary Fig. S8h, S8i), highlighting the safety of Aiphanol in vivo.

Collectively, we demonstrated that a naturally occurring stilbenolignan, Aiphanol, can directly target and inhibit VEGFR2 and COX2, thereby blocking angiogenesis and tumor growth (Fig. 1s). The structural characteristics of Aiphanol license a potent activity against angiogenesis through the cooperation among its stilbene unit, phenylpropane moiety, and dioxane bridge, which is distinct from the mechanisms of stilbenes or lignans. A combination of agents respectively inhibiting VEGFR2 and COX2 was shown to be effective in animal models of antiangiogenic therapy.^1^ Although inhibition of VEGFR2 mainly contributes to Aiphanol’s antiangiogenic function in vitro, the concomitant inhibition of COX2 in vivo may reprogram the proangiogenic microenvironment by declining the levels of PGE2 and VEGF. Our study supports Aiphanol as a potential antiangiogenic lead compound in cancer therapy.

## Supplementary information

Supplementary Materials

## Data Availability

All data generated or analyzed during this study are included either in this article or in the supplementary information files.
