# Correction: Aiphanol, a native compound, suppresses angiogenesis via dual-targeting VEGFR2 and COX2

**DOI:** 10.1038/s41392-022-00988-y

**Published:** 2022-04-30

**Authors:** Shanmei Chen, Junnan Feng, Chuanke Zhao, Lixin Wang, Lin Meng, Caiyun Liu, Shaoqing Cai, Yanxing Jia, Like Qu, Chengchao Shou

**Affiliations:** 1 Key Laboratory of Carcinogenesis and Translational Research (Ministry of Education/Beijing), Department of Biochemistry and Molecular Biology, Peking University Cancer Hospital & Institute, Beijing, China; 2 Key Laboratory of Natural and Biomimetic Drugs, School of Pharmaceutical Sciences, Peking University, Beijing, China; 3 Present Address: Key Laboratory of Molecular Pathology, The Affiliated Cancer Hospital of Zhengzhou University, Zhengzhou, China

**Keywords:** Tumour angiogenesis, Target identification

Correction to: *Signal Transduction and Targeted Therapy* 10.1038/s41392-021-00739-5, published online 03 December 2021

After online publication of the letter^1^, the author found two images in the supplement materials were used incorrectly. Additionally, there is an error in the chemical structure of Aiphanol in Figs. 1a and 1s that needs to be corrected. The correct data are provided as follows. The key findings of the article are not affected by these corrections. The original article has been corrected.

**Fig. 1:**
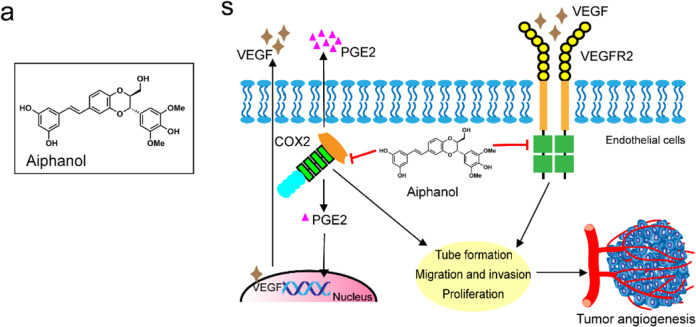
**a** Structure of Aiphanol. **s** The schematic representation of the mechanism

**Supplementary Fig. S1c:**
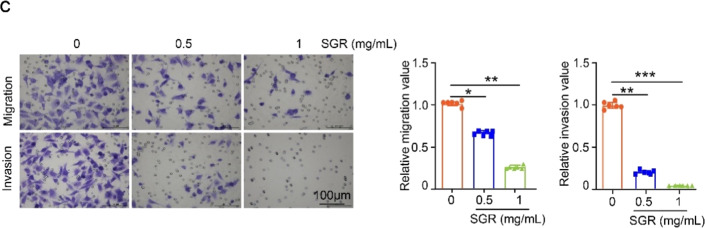
Transwell chamber analysis of SGR’s effects on the migratory and invasive abilities of HUVECs. Migrated or invaded cells were photographed and relative migration/invasion value was calculated (*n* = 6 per group). Scale bar, 100 μm. *P < 0.05; **P < 0.01; ***P < 0.001.

**Supplementary Fig. S4b:**
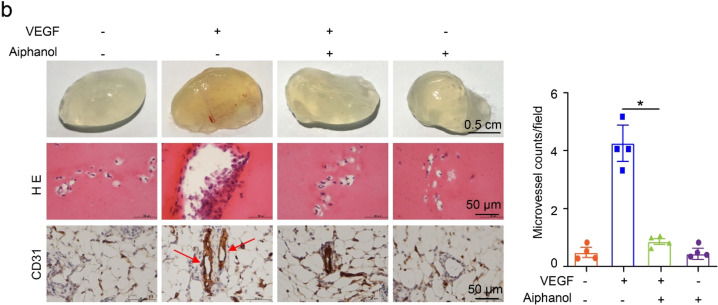
Matrigel plug assay of Aiphanol’s inhibition on the newly formed vessels. Paraffin-embedded sections of Matrigel plugs were stained with Hematoxylin and eosin (HE) or probed with anti-CD31 (brown). The numbers of neovessels (red arrows) were counted and compared (*n* = 4 per group). Scale bar, 0.5 cm (plugs) and 50 μm (sections). Data represented mean ± SEM. *P < 0.05.

## Supplementary information

Supplemental materials

Supplementary Fig S1c

Supplementary Fig S4b
