# Supplementary material for: Correction: Aiphanol, a native compound, suppresses angiogenesis via dual-targeting VEGFR2 and COX2
Source: Signal Transduct Target Ther. 2022 Apr 30;7:144. doi: 10.1038/s41392-022-00988-y (PMC9056504; doi:10.1038/s41392-022-00988-y)
Supplement: Supplementary file 1 — Supplemental materials [file 41392_2022_988_MOESM1_ESM.pdf]

Supplemental Materials

Aiphanol, a native compound, suppresses angiogenesis via dual-targeting VEGFR2 and COX2

Shanmei Chen [1], Junnan Feng [1#], Chuanke Zhao [1]*, Lixin Wang [1], Lin Meng [1], Caiyun Liu [1], Like Qu [1]*, Shaoqing Cai [2], Yanxing Jia [2], Chengchao Shou [1]*

1 Key Laboratory of Carcinogenesis and Translational Research (Ministry of Education/Beijing), Department of Biochemistry and Molecular Biology, Peking University Cancer Hospital & Institute, Beijing 100142, China.

2 Key Laboratory of Natural and Biomimetic Drugs, School of Pharmaceutical Sciences, Peking University, Beijing 100191, China.

# Current address: Key Laboratory of Molecular Pathology, The Affiliated Cancer Hospital of Zhengzhou University, Zhengzhou, 450008, China.

*Corresponding authors:

Chengchao Shou (cshou@vip.sina.com),

Chuanke Zhao (zhaochk@bjmu.edu.cn),

Like Qu (qulike99@163.com).

Supplementary information includes:

Materials and Methods

Supplementary Figures S1 - S8

Supplementary Table S1

Supplementary References

Materials and Methods

Cells and animals

Human umbilical vein endothelial cells (HUVECs) were obtained from undamaged section of fresh umbilical cords with collagenase digestion and verified by morphological characteristics of cobblestone-like pattern and the positive staining for CD31. HUVECs were maintained in EBM-2 medium with 2% FBS and used between passages 2-4 for all the experiments. Human microvascular endothelial cells (HMEC-1) and murine colon cancer cell line (MC38) were from the American Type Culture Collection (ATCC). Porcine aortic endothelial cells (PAEC) were gifted by Dr. Hetian Lei (Schepens Eye Research Institute of Massachusetts Eye and Ear, Harvard Medical School). HMEC-1 and PAEC were cultured in DMEM medium supplemented with 10% fetal bovine serum (FBS). MC38 was cultured in RPMI-1640 medium supplemented with 10% FBS.

Female C57/BL6 mice were purchased from HFK Bioscience (Beijing, China) and raised by the Experimental Animal Facility of Peking University Cancer Institute.

Chemical reagent

Sarsaparilla (SGR) was obtained from Ben Cao Fang Yuan Pharmaceutical (Beijing, China). The extraction of SGR was completed in collaboration with Professor Cai Shaoqing's laboratory at the School of Pharmaceutical Sciences, Peking University and reported previously. [1] Lyophilized SGR was dissolved in PBS and diluted to indicated concentrations in medium. Aiphanol was synthesized by Huatian Inc. (Chengdu, China) and Professor Jia Yanxing's laboratory at the School of Pharmaceutical Sciences, Peking University, with purity greater than 99.9% by high-performance liquid chromatography (HPLC). Carboxyfluorescein (FAM)-conjugated Aiphanol was provided by Chengdao Biotechnologies (Beijing, China) with purity greater than 99% by HPLC. Resveratrol, Naringenin, Kaempferol and Celecoxib were purchased from MedChemExpress (Shanghai, China), with the respective purities greater than 99.7%, 98.7%, 99.6%, and 99.5% by HPLC. Compounds were dissolved in dimethyl sulfoxide (DMSO), and diluted to the indicated working concentrations in medium. The concentration of DMSO in working solution was less than 1 ‰.

Recombinant human VEGF165 and Recombinant human bFGF were purchased from MedChemExpress (Monmouth Junction, USA). Animal-Free recombinant human EGF was obtained from Peprotech (Cranbury, USA). Bevacizumab was purchased from Roche (Basel, Switzerland). The Growth Factor Reduced Matrigel was obtained from BD Biosciences (Franklin Lakes, USA). Recombinant human COX2 and VEGFR2 were purchased from Sino Biological (Beijing, China).

Antibodies

Anti-caspase3 (#9668), anti-cleaved caspase-3 (#9661), anti-PARP (#9532), anti-P53 (#2527), anti-Bax (#5023), anti-VEGFR2 (#2479), anti-phospho-VEGFR2 (Tyr^{1175}) (#2478), anti-FAK (#71433), anti-AKT (#4691), anti-phospho-AKT (Ser^{473}) (#4060), anti-ERK (#4696), anti-phospho-ERK (Tyr^{202}/Tyr^{204}) (#4370), anti-CD31 (#77699), Alexa Fluor 488-conjugated secondary antibody (#8494), and Alexa Fluor 594-conjugated secondary antibody (#8889) were purchased from Cell Signaling Technology (Danvers, USA). Anti-caspase9 (AC062) was from Beyotime (Shanghai, China). Anti-phospho-FAK (Tyr^{397}) (sc-11765-R) was from Santa Cruz (Dallas, USA). Anti-VEGFR1 (TA303515) was obtained from OriGene (Rockville, USA). Anti-β-tubulin (K200059M) was from Solarbio (Beijing, China). Anti-VEGF was purchased from Proteintech (Rosemont, USA). Anti-phosphoserine/threonine and anti-phosphotyrosine were obtained from BD Biosciences (Shanghai, China). Anti-GAPDH, anti-Factor VIII and horseradish peroxidase (HRP)-labeled secondary antibodies were purchased from ZSGB Biotechnology (Beijing, China).

Evaluation of cellular intake of Aiphanol in cells by immunofluorescence staining

HUVECs or MC38 cells were incubated with FAM-labeled Aiphanol (15 μM) for 6 h. For live cell analysis, the cells were counterstained with 5 μM Hoechst 33342 for 10 min. For fixed cell analysis, the cells were fixed with 4% paraformaldehyde for 20 min, followed by permeabilization with 0.1% Triton X-100 for 5 min and blocked with 5% goat serum for 1h at room temperature. Anti-β-tubulin was applied to the cells overnight at 4°C. After washing with PBST, the cells were probed with Alexa Fluor 594-conjugated secondary antibody and counterstained with 4,6-diamidino-2-phenylindole (DAPI). Images were captured under the Leica TCS-SP2 confocal microscopy (Heidelberg, Germany).

Tube formation assay

In vitro angiogenesis was performed as previous reported.[2] Briefly, the 96-well plate pre-coated with 50 μL growth factor reduced Matrigel was incubated at 37°C for 1 h to solidify as base. Cells were resuspended in serum-free medium containing 0.1% BSA. Each well with polymerized Matrigel was added with 100 μL cell suspension containing 15,000 cells with or without 20 ng/mL of growth factors and indicated chemicals. After 6 h, the tubes were photographed under the microscope and quantified with by the Angiogenesis Analyzer plugin in Image J.

Cell growth assay

The cells were seeded in 96-well plates at a density of 4,000 cells/well, then treated with vehicle or indicated concentrations of SGR or Aiphanol for 24 h. CCK8 reagent from Dojindo (Kumamoto, Japan) was added to wells for 1 h incubation. The amount of formazan salt was determined by measuring the optical density at 450

nm using a Bio-Rad microplate reader. Alternatively, cell confluence rates were detected every 24 h by the CloneSelect Imager from Molecular Devices.

Colony formation assay

For the plate colony formation assay, 1,000 MC38 cells were seeded in 6-well plates and cultured for 12 d. Colonies were fixed in cold methanol, stained with 0.1% crystal violet, and counted under the microscope. For the soft-agar colony formation assay, 6-well plates were pre-coated with RPMI 1640 medium containing 0.6% agarose as base. After solidification, 1,000 MC38 cells resuspended in RPMI 1640 medium containing 0.35% agarose were seeded. Plates were incubated for 14 d and colonies were counted under microscope.

Apoptosis assay

After treatment with 30 μM Aiphanol for 24 h, HUVECs were harvested and stained with AnnexinV-FITC/PI Apoptosis Detection Kit from Dojindo according to the manufacturer's protocol. Apoptotic cells were measured by flow cytometry (BD Biosciences, USA).

Cell-cycle analysis

After treatment with 30 μM Aiphanol for 24 h, HUVECs were harvested by trypsinization and fixed in 75% ethanol overnight at 4°C. The cells were stained with PI mixture and analyzed by flow cytometry with ModFit LT 3.0 (Verity Software House, USA).

Wound Healing assay

HUVECs were plated in 6-well plates until the confluence reached 90%. A straight wound line was drawn across the attached cell layer. After washing with PBS twice to remove remaining FBS and floating cells, the cells were cultured in serum-free medium and exposed to SGR or Aiphanol. Pictures of the marked gaps were taken after 24 h. The wound gaps were digitally quantified using Image Pro Plus software.

Transwell migration and invasion assay

The migrated HUVECs suspension (40,000 cells in 200 μL) or invaded cells suspension (50,000 cells in 500 μL) was treated with SGR or Aiphanol and seeded into the upper well of transwell inserts (Corning, USA) for 24 h. The lower well was added with EBM-2 medium containing 2% FBS as chemotactic attractors. The cells that have penetrated to bottom side of the transwell membranes were fixed with methanol and stained with 0.1% crystal violet. 5-6 random fields were captured under microscope and the numbers of migrated or invaded cells were counted.

Western Blot

The cells were homogenized in RIPA buffer containing 50 mM Tris-HCl (pH 7.5), 20 mM NaF, 1 mM EDTA,

1 mM Na_3VO_4, 1% NP-40, 0.5% sodium deoxycholate, 0.1% SDS, 2 mM Dithiothreitol, and 1 x protease inhibitor cocktail from Roche. Protein concentration was quantified by the BCA kit (Thermo Fisher Scientific, USA). Cell lysates (30 μg per sample) were subjected to SDS–PAGE and transferred onto nitrocellulose membranes. After blocking with 5% milk in 0.1% Tween 20/TBS (TBST) for 1 h at room temperature, the nitrocellulose membranes were probed with indicated antibodies for 12 h at 4°C. The protein bands were visualized with Enhanced Chemiluminescence Detection Kit-HRP (Biological Industrials, Israel).

Enzyme linked immunosorbent assay (ELISA)

Levels of VEGF in the conditioned medium or MC38 tumor tissues were measured by VEGF DuoSet from R&D System (Shanghai, China). Levels of PGE2 in the conditioned medium or plasma of mice were analyzed by PGE2 detection kit from Cloud-Clone Corp (Wuhan, China) according to the manufacturer's instructions. Optical densities at 450 nm were measured with a Bio-Rad microplate reader.

Cell-ELISA

HUVECs were plated in 96-well plates and exposed to Aiphanol (30 μM) for 0.5 to 24 h. After fixation with 2.5% glutaraldehyde and permeabilization with 0.5% TritonX-100, anti-phosphoserine/threonine or anti-phosphotyrosine antibody was added to the wells and incubated for 2 h. After washing with TBST, the plates were incubated with HRP-conjugated anti-mouse-IgG for 1 h. After washing for three times, plates were incubated with 3,3',5,5'-Tetramethylbenzidine (TMB) solution (Cloud-Clone Corp) and stop solution (1N H_2SO_4). Optical densities at 450 nm were quantified using a Bio-Rad microplate reader and represented as relative levels of phospho-proteins.

COX enzymatic activity assay

The COX Fluorescent Screening Assay Kit from Cayman Chemical (Ann Arbor, USA) was utilized according to the manufacturer's instructions to examine the effect of Aiphanol on the activities of COX-1/COX-2 isozymes. The final product resorufin with high fluorescent was analyzed with an excitation wavelength of 530-540 nm and an emission wavelength of 585-595 nm.

Kinase profiler^{TM} assay

The kinase profiler^{TM} assay was performed by Eurofins Cerep SA (Celle L'Evescault, France). Aiphanol (30 μM) was co-incubated with different kinases in the reaction buffer containing 8 mM MOPS (pH 7.0), 0.2 mM EDTA, 0.1% β-mercaptoethanol, 10 mM Magnesium Acetate and γ-^{32}P-ATP. The reaction was initiated by the addition of the Mg/ATP mix. After incubation for 40 min at room temperature, the reaction was stopped by the addition of phosphoric acid. Reaction mix (10 μL) was then spotted onto a P30 filtermat for scintillation

counting. The results were calculated using the following formula: (Mean of Sample Counts – Mean of Blank Counts)/Mean of Control Counts.

Molecular docking

Crystal structure of the human COX2 protein (PDB ID: 5IKV) and its selective inhibitor, flufenamic acid, was retrieved from the protein databank. Crystal structure of the human VEGFR2 protein (PDB ID: 4ASE) and its selective inhibitor, Tivozanib, was retrieved as above. The Protein Preparation Wizard Panel was used to modify the three-dimensional (3D) model of COX2 or VEGFR2, including dehydrating, hydrogenating, repairing the missing residue, optimizing the structure, and minimizing the energy (OPLS2005 force field, 0.30 Å RMSD). The box size was 20 Å × 20 Å × 20 Å. 2D format of Aiphanol was dealt with LigPrep module for energy minimization to output the corresponding 3D structure. The sophisticated mode of Glide module was used for molecular docking, that is, the receptors and ligands were docked with each other through geometric and energy matching. The binding patterns of the COX2-Aiphanol and VEGFR2-Aiphanol were plotted in 2D and 3D upon the output.

Microscale thermophoresis (MST) Measurement

COX2 was labeled in the supplied labeling buffer for 30 min using Monolith Protein Labeling Kit RED-NHS from NanoTemper (Munich, Germany). Then COX2 was diluted with buffer containing 50 mM Tris-HCl (pH 8.0), 0.05% Tween, 1 nM Hemin. The binding affinity of COX2-Aiphanol was determined with the Monolith NT.115 instrument from NanoTemper. The dissociation constant (Kd) of the interaction was calculated by fitting the data using MST standard fit algorithm in the MO. Affinity Analysis Software from NanoTemper.

Binding kinetics analysis with Surface plasma resonance (SPR)

The SPR experiment was performed with a Biacore T200 on CM5 sensor chips from GE Healthcare (Seattle, USA). After activation of CM5 sensor chip, VEGFR2 was diluted to 25 μg/mL in NaAc (pH 5.0) and immobilized at the flow velocity of approximately 10 μL/min for 30 min to conduct with the standard amine coupling procedure. Aiphanol was dissolved in DMSO and diluted in buffer containing 10 mM MOPS (pH 7.2), 150 mM NaCl, 1 mM $MgCl_2$ and 0.05% Surfactant P20, followed by injection over the functionalized surface at a flow rate of 20 μL/min. The sensorgrams and the kinetic parameters were determined by Biacore S200 evaluation software 2.1.

BioLayer Interferometry (BLI)

The binding between VEGF and VEGFR2 protein were detected using the Octet RED96 System from Forté Bio (Menlo Park, USA). VEGFR2 was conjugated to EZ-Link NHS-PEG12-Biotin and diluted to 10 μg/mL by

HBS-EP+ buffer (GE Healthcare). Streptavidin sensors were pre-wetted by dipping into HBS-EP+ buffer for 15 min and loaded with biotinylated VEGFR2 for 720 s to establish a baseline signal and diluted with HBS-EP+ buffer for 180 s to remove non-specific adsorption. Then the sensors were immersed into different concentrations of VEGF solution to associate for 300 s and dissociate for 360 s in HBS-EP+ buffer. Sensorgrams were obtained at each concentration and the Kd was calculated by the FortéBio Data Analysis 12.0. To evaluate the interfering effect of Aiphanol on VEGF-VEGFR2 interaction, 2x Kd concentration of VEGF and different concentrations of Aiphanol were mixed at same volume. Bevacizumab (Bev) was used as the positive control to inhibit VEGF-VEGFR2 association. Then the immobilized sensors were dipped into the mixture for association and returned to HBS-EP+ buffer for dissociation. Finally, data analysis was performed using a reference subtraction and aligned using baseline signal.

ADP-Glo™ assay

The ADP-Glo™ Kinase Kit from Promega (Madison, USA) was used to evaluate Aiphanol's effect on VEGFR2 kinase activity. The kinase buffer mixture contained different concentrations of Aiphanol, 1.5 ng/μL VEGFR2, 0.2 μg/μL poly (4:1 Glu, Tyr) peptide, and 50 μM ATP. The reaction was started by addition of ATP at 37°C for 1 h. 5 μL ADP-Glo™ reagent was added to each well to stop the reaction and incubated at room temperature for 40 min. Finally, 10 μL kinase detection reagent was added to the wells and incubated for 30 min to generate luminescent signals, which were measured under an automated microplate reader and the dose-response curve was fitted.

siRNA interference assay

Small interfering RNAs (siRNA) were synthesized by GenePharma (Suzhou, China). The siRNA sequences were as follows: small interference negative control (siNC) sequences: 5'-UUCUCCGAACGUGUCACGUTT-3'. COX2 siRNA #1 5′-GCAGCUUCCUGAUUCAAAUTT-3′, COX2 siRNA #2 5′-AAC UGC UCA ACA CCG GAA UTT-3′, COX2 siRNA #3 5′-AGACAGATCATAAGCGAGGA-3′. VEGFR1 siRNA #1 5'- GCCGGAAGUUGUAUGGUATT-3', VEGFR1 siRNA #2 5'-CGUGGCUACUCGUUAAUUATT-3', VEGFR1 siRNA #3 5'-GCUGUACCUACUUCAAAGATT-3'. VEGFR2 siRNA #1 5'- CCACCAAUUCCCGUUGGUUTT-3', VEGFR2 siRNA #2 5'-GGUAAAGAUUGAUGAAGAA-3', VEGFR2 siRNA #3 5'-GAUGUAAGCUCUCCUCCAUTT-3'. HUVECs seeded 6-well plates were transfected with 35 nM siRNA plus lipofectamine RNAiMAX from Invitrogen (Carlsbad, USA) for 48 h.

Transgenic zebrafish model

Transgenic zebrafish model of angiogenesis was performed by Hunter Biotechnology (Hangzhou, China) following established procedures with some modifications. [3] Two concentrations of Aiphanol (2.8, 8.3 μM) were set according to the results of the pre-assay. Drugs were injected into the yolk sac of zebrafish **(for)** 48 h after fertilization by the microinjection method. After incubation for 24 h at 28°C, fluorescence of the subintestinal vessels (SIVs) was observed and photographed using a multi-purpose zoom microscope system (Nikon, Japan). Image analysis was performed by calculating the SIVs area via Nikon NIS-Elements D 3.10 Advanced image processing software.

Matrigel plug assay

Matrigel plug angiogenesis assay was performed following previously reported protocol with some modifications. [4] C57BL/6 mice were randomly divided into four groups: PBS, VEGF, Aiphanol, and VEGF combined with Aiphanol. The mice (n = 4 per group) were subcutaneously injected with 400 μL Matrigel containing VEGF (500 ng/mL) with or without 15 μM Aiphanol. The mice were sacrificed after 14 d and Matrigel plugs were removed and photographed. The plugs were fixed in 10% buffered formaldehyde, embedded in paraffin and sectioned. The 4 μm-thick sections were stained with anti-CD31. CD31-positive vessels in each 200x magnification were scored and quantified.

Mouse aortic ring assay

The procedures were reported previously. [5] The 48-well plates were coated with 120 μL of Matrigel per well and polymerized at 37°C for 1 h. Aortas harvested from 7-week-old C57BL/6 mice were sliced into 1 mm thick rings. The rings were placed on the polymerized Matrigel and sealed with 60 μL Matrigel for 3 h to allow Matrigel to be polymerized firmly. 500 μL serum-free EBM-2 medium containing VEGF (20 ng/mL) with or without indicated concentrations of Aiphanol was added into the wells. Bevacizumab (Bev, 0.5 mg/mL) was used as the positive control. After 6 d, the microvessel area was photographed and analyzed with Image J.

Chicken embryo chorioallantoic membrane (CAM) assay

CAM assay was performed following previously reported procedures with some modifications. [2] Briefly, fresh fertilized white leghorn chicken eggs from Boehringer Ingelheim Vital Biotechnology (Beijing, China) were swabbed by 1% bromogeramine and incubated at 37°C with 60% humidity. On the third day of embryo development, the whole egg contents were gently transferred into sterilized dishes and incubated at 37°C. Glass cellulose filters, which were soaked with VEGF (20 ng/mL) with or without indicated concentrations of Aiphanol, were applied to CAMs. Bevacizumab (Bev, 0.5 mg/mL) was used as the positive control. Then the embryos were cultured for 48 h. Vascular response to drugs was evaluated by counting the number of blood

vessels within the defined area of the filters under stereomicroscope (Olympus, Japan).

MC38 syngeneic mouse model

C57BL/6 mice were subcutaneously injected with 7 x 10^5 MC38 cells which were resuspended in PBS containing 50% growth factor reduced Matrigel. Aiphanol was dissolved in the solvent (10% DMSO, 40% PEG300, 3% Tween-80, 47% saline). Upon tumors reaching 100 mm^3, mice were administered orally with 100 μL of Aiphanol (30 mg/kg for single-dosage experiment, 5 and 30 mg/kg for double-dosage experiment) or solvent control once daily. Bevacizumab (Bev, 5 mg/kg) was injected through tail vein as the positive control every four days in the double-dosage experiment. Tumor volume was measured thrice weekly and calculated as $width^2$ x length/2. Mice were sacrificed after 12 d (for single-dosage experiment) or 8 d (for double-dosage experiment), the tumors were resected and weighed.

Immunohistochemistry (IHC)

The tumors from single-dosage experiment were resected and fixed in 10% buffered formaldehyde for 48 h. Tumors were paraffin-embedded, sectioned (4 μm thickness), deparaffinized and hydrated. Antigen retrieval was performed by autoclave heating in Tris-EDTA buffer (pH 9.0) for 15 min. Sections were then quenched with 3% hydrogen peroxide for 10 min to block endogenous peroxidase activity and blocked with normal goat serum for 40 min at room temperature. Primary antibodies were applied to the sections overnight at 4°C. After probing with secondary antibodies, the peroxidase reaction was carried out with Liquid DAB Substrate (GeneTech, USA). Finally, slides were counterstained with hematoxylin (ZSGB Biotechnology). The intensity of IHC staining was quantified by the IHC Profiler plugin in Image J.

Frozen sections

The resected tumors from single-dosage experiment were flash-frozen in liquid nitrogen and immediately embedded in optimal-cutting-temperature (OCT) compound (Sakura) for sectioning with the freezing microtome (Leica, Germany) by the pathologist. Frozen sections were subjected to immunofluorescence staining analysis of CD31 expression.

Statistical Analysis

Graph Pad Prism 6.0 software was used to perform an ANOVA test and the Student's two-tailed unpaired t-test. Values are presented as means ± Standard Error of the Mean (SEM). $P < 0.05$ was considered statistically significant. *$P < 0.05$; **$P < 0.01$; ***$P < 0.001$; n.s., no significance.

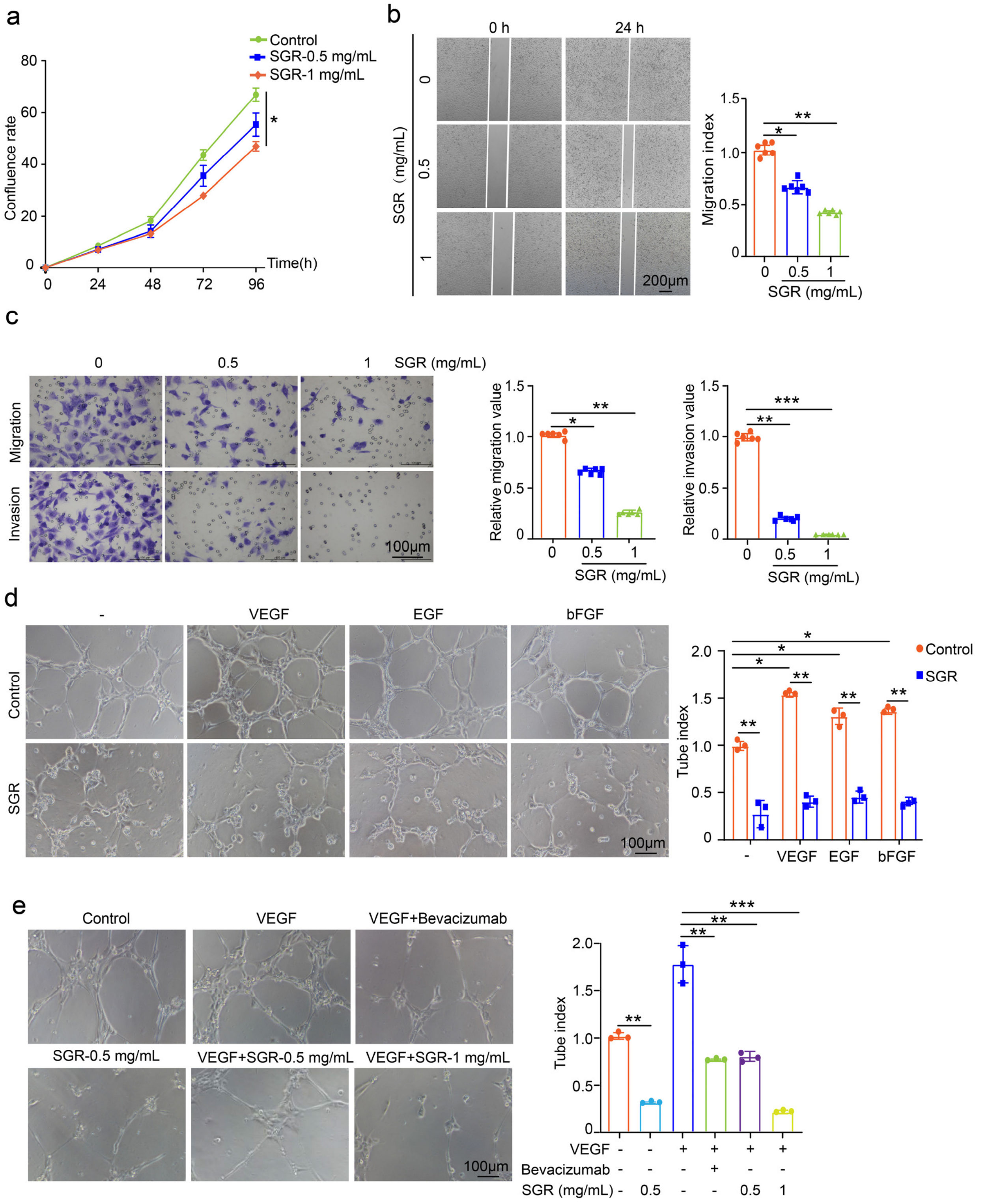

Supplementary Fig S1. *Sarsaparilla* (SGR) inhibits angiogenesis *in vitro*. (a) HUVECs were treated with different concentrations of SGR for indicated time and cell confluence rates were analyzed every 24 h by the CloneSelect Imager (n = 4 per group). (b) Wound healing assay was performed to examine the effects of SGR on the motility of HUVECs. The widths of the injury lines were captured and quantified (n = 6 per group). Scale bar, 200 µm. (c) Transwell chamber analysis of SGR's effects on the migratory and invasive abilities of

HUVECs after Aiphanol treatment for 24 h. Migrated or invaded cells were photographed and relative migration/invasion value was calculated (n = 6 per group). Scale bar, 100 μm. (d) Effects of SGR on tube formation of HUVECs induced by VEGF, EGF or bFGF (all at 20 ng/mL). Tubular structures were photographed and the tube index was quantified (n = 3 per group). Scale bar, 100 μm. (e) Dose-dependent effect of SGR on VEGF-induced tube formation of HUVECs. Bevacizumab (0.5 mg/mL) was used as positive control. Tubular structures were photographed and tube index was quantified (n = 3 per group). Scale bar, 100 μm. Data represented mean ± SEM. * $P < 0.05$; ** $P < 0.01$; *** $P < 0.001$.

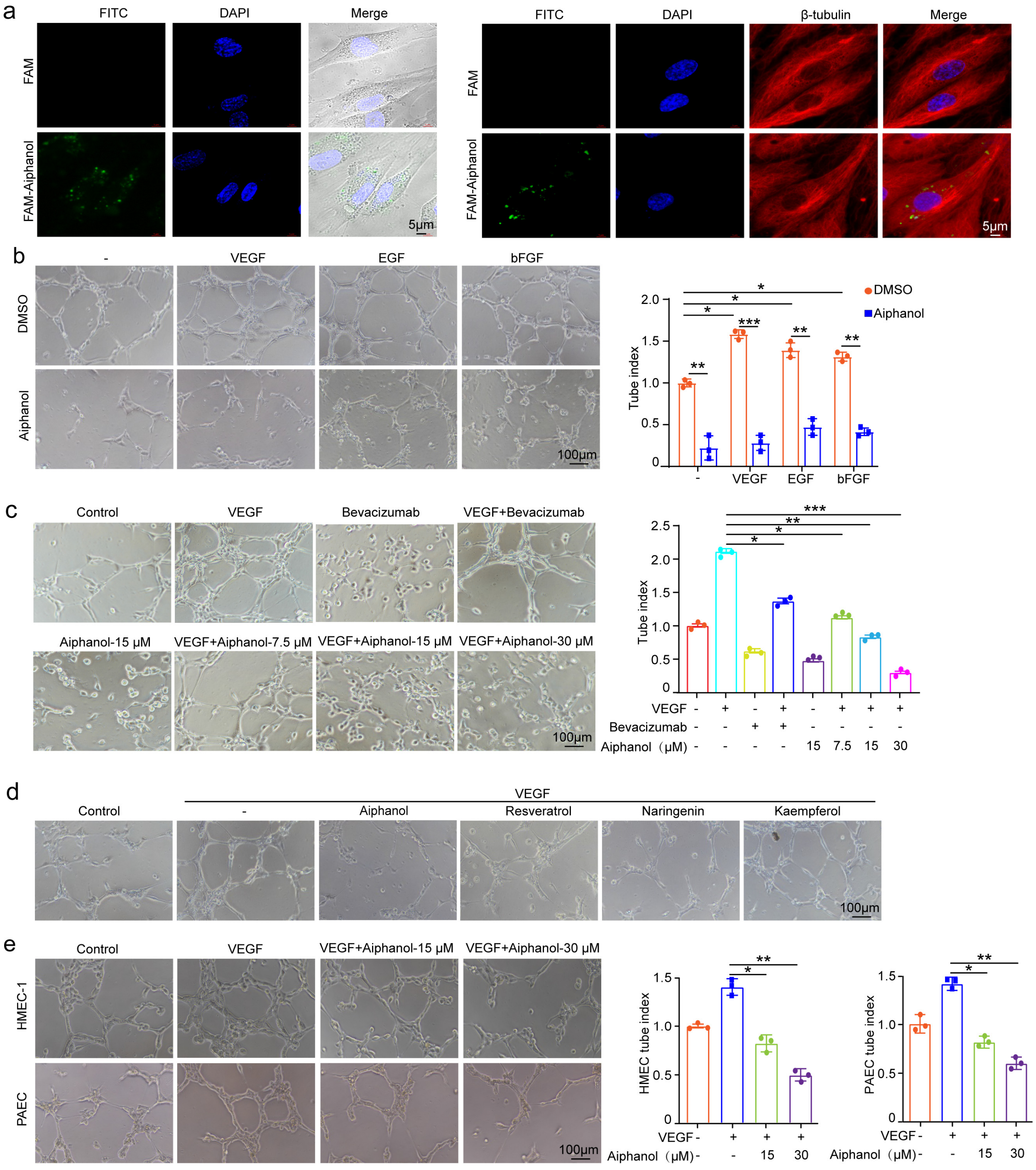

Supplementary Fig S2. Aiphanol inhibits angiogenesis *in vitro*.

(a) Entry and distribution of Aiphanol in HUVECs. Cells were treated with 15 μM FAM-Aiphanol (green) for 6 h. For live cell analysis (left panel), the cells were stained with Hoechst 33342 (blue). For fixed cell analysis (right panel), the cells were counterstained with anti-β-tubulin (red) and DAPI (blue). Scale bar, 5 μm. (b) Effects of Aiphanol on tube formation of HUVECs induced by VEGF, EGF or bFGF (all at 20 ng/mL). Tubular structures were photographed and the tube index was quantified (n = 3 per group). Scale bar, 100 μm. (c)

Dosing effects of Aiphanol on VEGF-induced tube formation of HUVECs (n = 3 per group). Bevacizumab (0.5 mg/mL) was used as positive control. Scale bar, 100 μm. (d) Representative photos showing the effects of Aiphanol, Resveratrol, Narigenin, and Kaempferol (all at 30 μM) on VEGF-induced tube formation of HUVECs. Scale bar, 100 μm. (e) Effects of Aiphanol on VEGF-induced tube formation of HMEC-1 and PAEC (n = 3 per group). Scale bar, 100 μm. Data represented mean ± SEM. * $P < 0.05$; ** $P < 0.01$; *** $P < 0.001$.

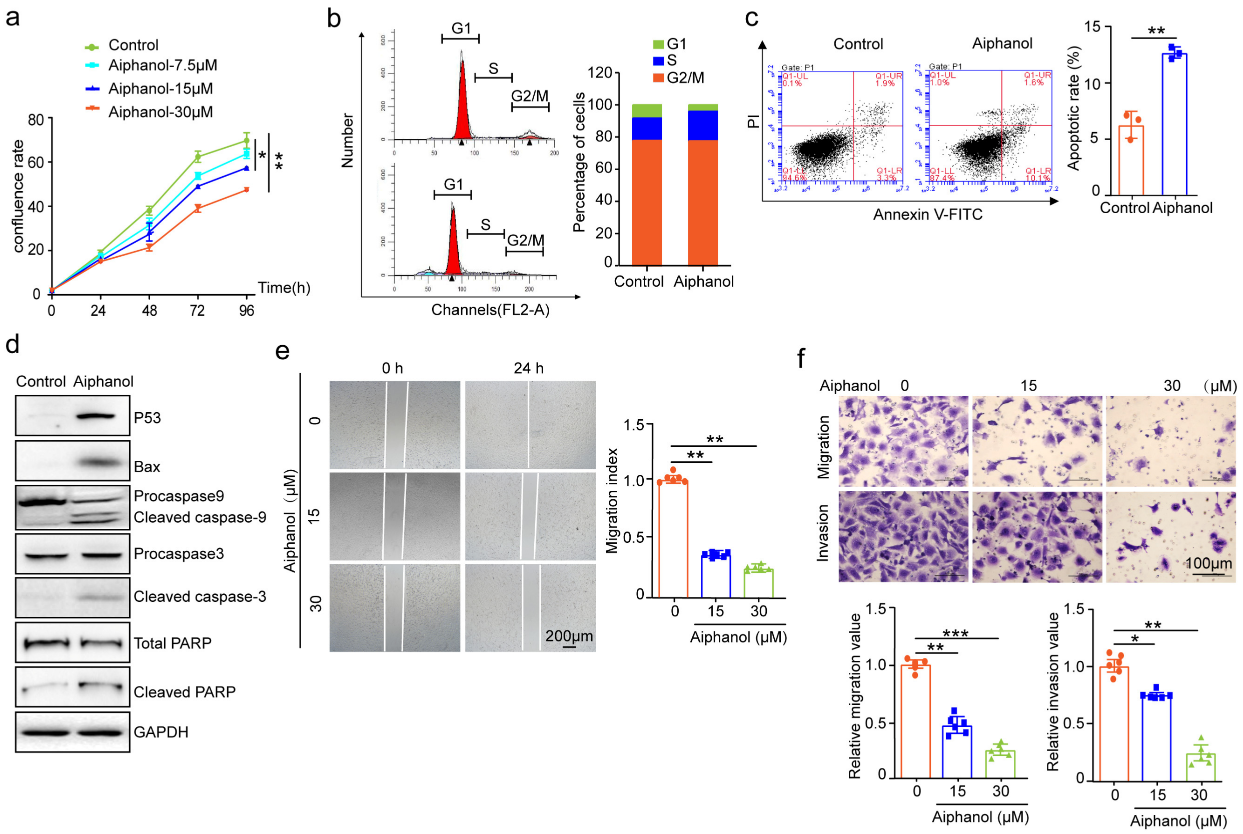

Supplementary Fig. S3. Aiphanol inhibits the viability and motility of HUVECs.

(a) HUVECs were treated with different doses of Aiphanol for indicated time and cell confluence rates were analyzed every 24 h by the CloneSelect Imager (n = 4 per group). (b) Cell cycle analysis of HUVECs after treatment with 30 μM Aiphanol for 24 h. (c) Annexin V/PI-staining of apoptotic HUVECs treated with 30 μM Aiphanol or control for 24 h (n = 3 per group). (d) Western blot analysis of indicated proteins in HUVECs treated with 30 μM Aiphanol or control for 48 h. GAPDH as a loading control. (e) Wound healing assay of Aiphanol's effect on the migration of HUVECs (n = 6 per group). Scale bar, 200 μm. (f) Transwell chamber analysis of Aiphanol's effects on the migratory and invasive abilities of HUVECs after Aiphanol treatment for 24 h. Migrated or invaded cells were photographed and relative migration/invasion values were calculated (n = 6 per group). Scale bar, 100 μm. Data represented mean ± SEM. *P < 0.05; **P < 0.01; ***P < 0.001.

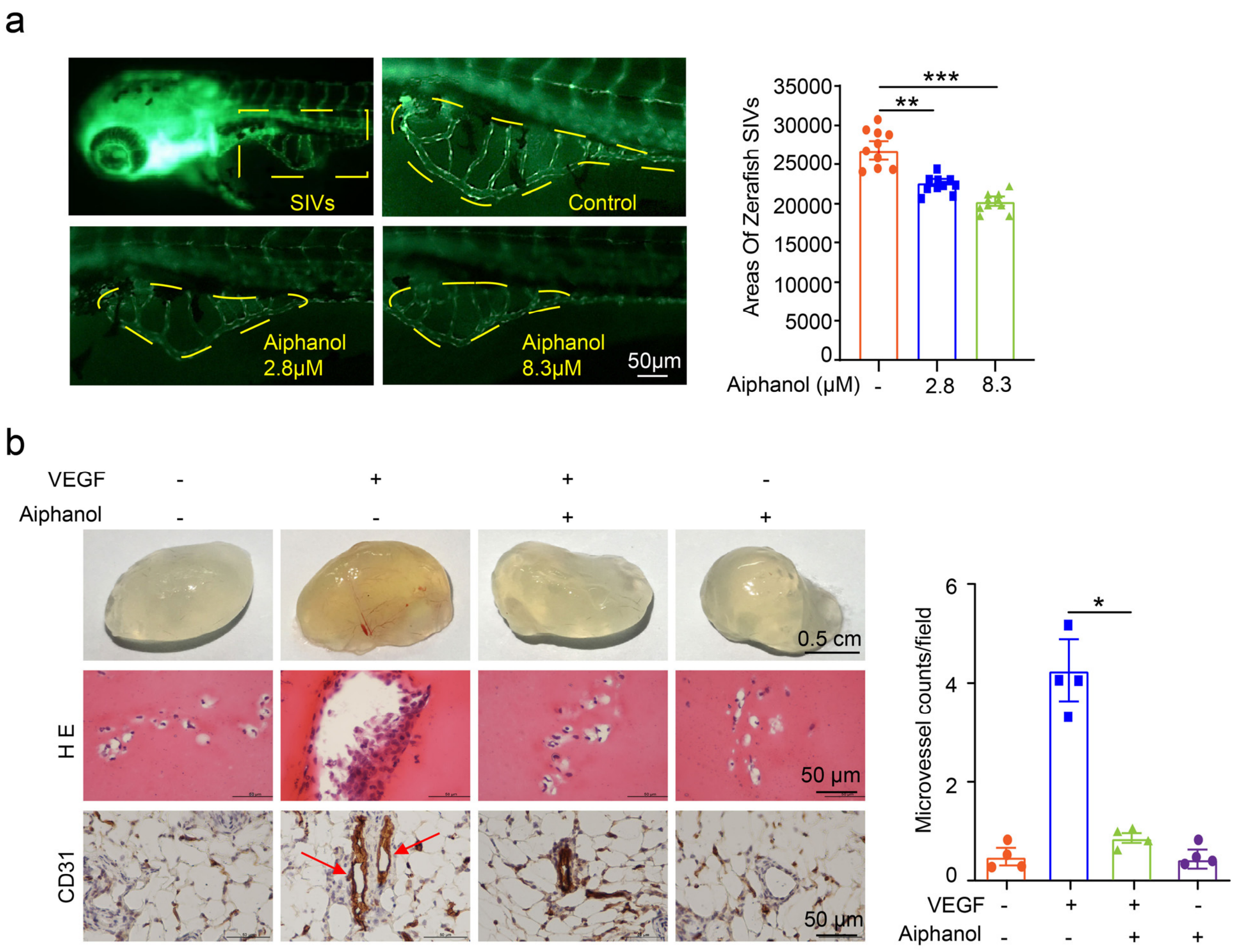

Supplementary Fig S4. Aiphanol inhibits angiogenesis *in vivo*.

(a) The zebrafish model was used to assess Aiphanol's inhibitory effect on the formation of intestinal venous plexus. After treatment for 48 h, the subintestinal vessels (SIVs) were photographed and fluorescence of the SIVs area was quantified and compared (n = 10 per group). Scale bar, 50 μm. (b) Matrigel plug assay of Aiphanol's inhibition on the newly formed vessels. Paraffin-embedded sections of Matrigel plugs were stained with Hematoxylin and eosin (HE) or probed with anti-CD31 (brown). The numbers of neovessels (red arrows) were counted and compared (n = 4 per group). Scale bar, 0.5 cm (plugs) and 50 μm (sections). Data represented mean ± SEM. * $P < 0.05$; ** $P < 0.01$; *** $P < 0.001$.

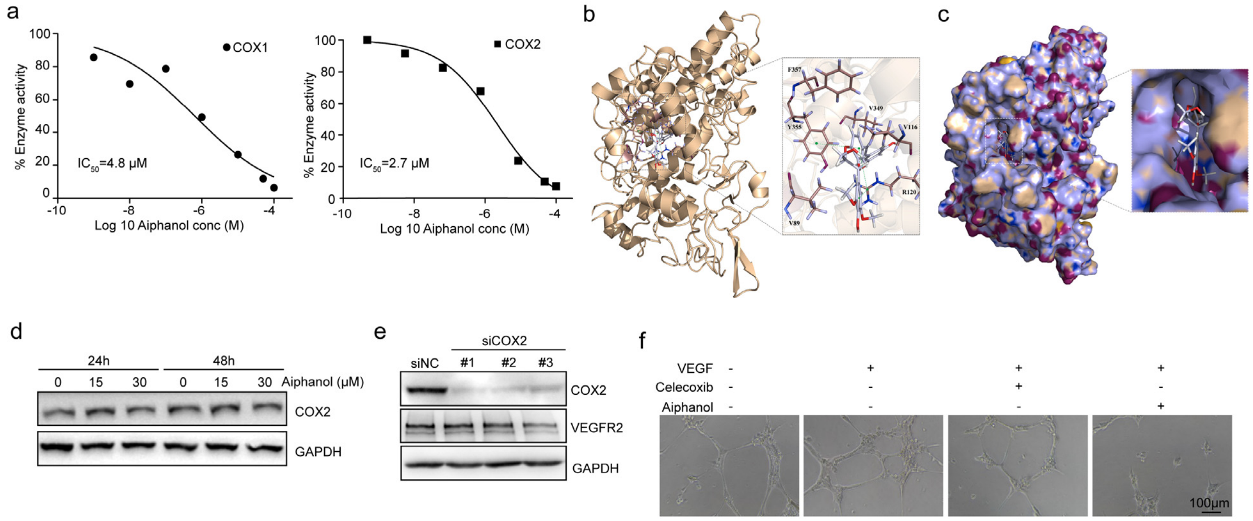

Supplementary Fig S5. Aiphanol inhibits COX2 activity.

(a) IC_{50} measurement of Aiphanol-inhibited COX1 and COX2 activities through fluorescent screening assay. (b, c) Docking prediction of Aiphanol-COX2 interaction. 3D representation (b) and surface representation (c) of Aiphanol in the COX2 binding sites were shown. Aiphanol could form one π-π, two cationic-π and multiple hydrophobic interactions with COX2. The benzene ring of benzo-dioxins could form one π-π interaction with Tyr355 (Y355) and one cationic -π interaction with Arg120 (R120) which became another cationic-π action with the m-dimethoxyphenyl benzene ring. In addition, aiphanol shared hydrophobic contacts through Val89 (V89), Val116 (V116), Val349 (V349), Phe357 (F357) of COX2. (d) Western blot analysis of COX2 in HUVECs treated with Aiphanol. (e) HUVECs were transfected with COX2 siRNA or scrambled siRNA for 48 h, and Western blot was used to determine COX2 and VEGFR2 levels. (f) Representative photos showing the effects of Aiphanol and Celecoxib (both at 30 μM) on tube formation of HUVECs.

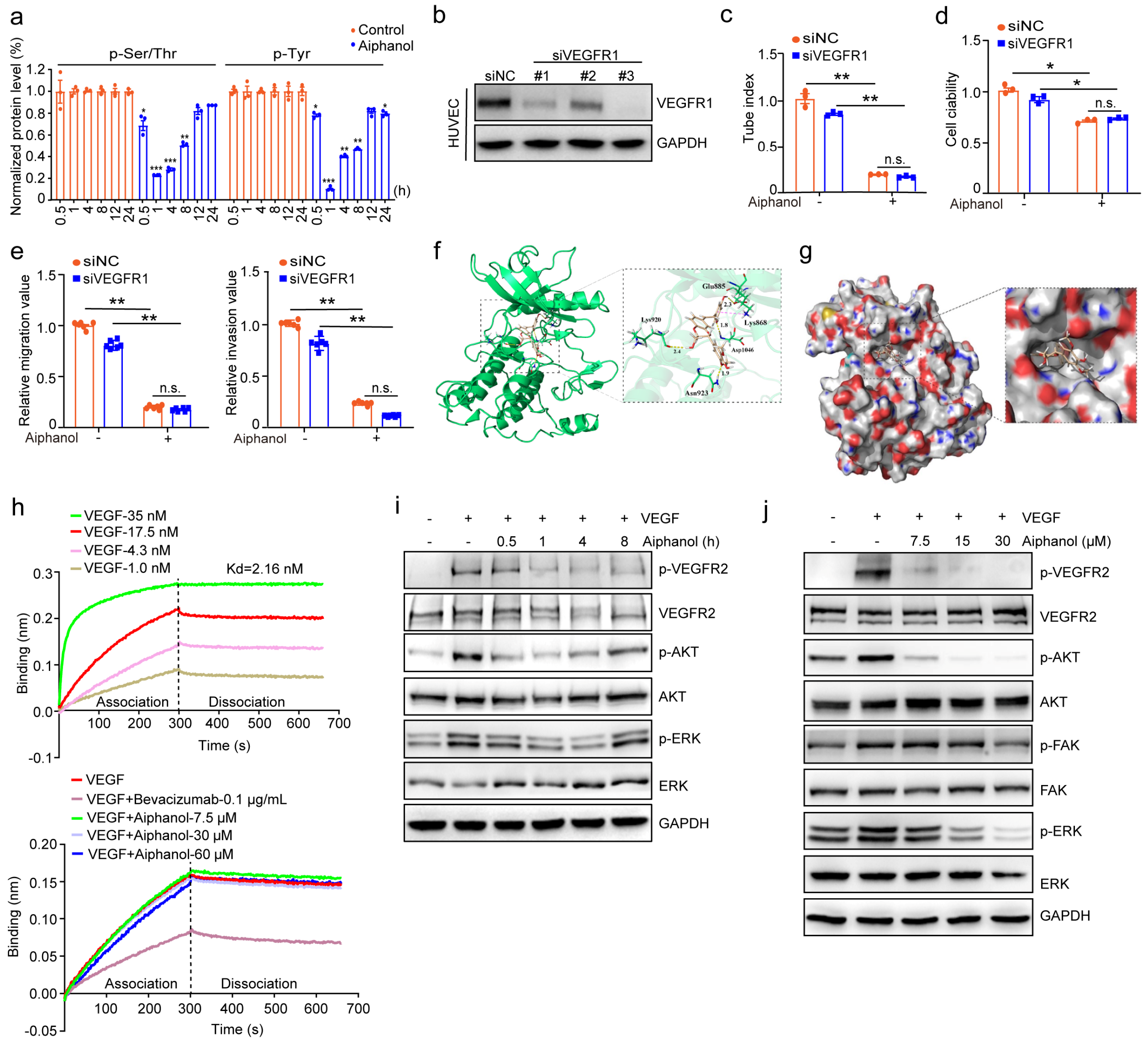

Supplementary Fig S6. Aiphanol inhibits the activities of kinases associated with angiogenesis and VEGFR2 signaling pathway.

(a) HUVECs were exposed to 30 μM Aiphanol for 0.5 to 24 h, then the global phosphorylation profiles of serine/threonine (Ser/Thr) and tyrosine (Tyr) were measured by ELISA (n = 3 for each time point). (b) HUVECs were transfected with VEGFR1 siRNA or scrambled siRNA for 48 h. Western blot was performed to evaluate the level of VEGFR1. (c) Effects of VEGFR1 silencing on Aiphanol-inhibited tube formation of HUVECs. Cells were transfected with VEGFR1 siRNA or scrambled siRNA for 36 h, followed by tube formation assay for 6 h with or without treatment of 30 μM Aiphanol (n = 3 per group). (d) Effects of VEGFR1 silencing on Aiphanol-inhibited viability of HUVECs. Cells were transfected with VEGFR1 siRNA or scrambled siRNA for 36 h, followed by treatment of 30 μM Aiphanol for 24 h. Cell viability was analyzed by CCK8 assay (n = 3 per group). (e) Effects of VEGFR1 silencing on Aiphanol-inhibited migration and

invasion of HUVECs. Cells were transfected with VEGFR1 siRNA or scrambled siRNA for 36 h, followed by transwell chamber assays for 24 h with or without treatment of 30 μM Aiphanol (n = 6 per group). (f, g) Docking model of Aiphanol-VEGFR2 interaction. 3D representation (f) and Surface representation (g) were shown. Aiphanol could form four hydrogen bonds and one salt bridge with VEGFR2. The OH on resorcinol acted as the donor and acceptor respectively to form hydrogen bonds with Glu885 and Asp1046. The OH on the alcohol hydroxyl treated as the donor could form a hydrogen bond with Lys920. The O on the methoxy moiety acted as the receptor to form a hydrogen bond with Asn923. Moreover, the benzene ring on resorcinol could form a salt bridge with Lys868. (h) BLI analysis of VEGFR2-VEGF interaction. After the kinetic binding analysis of VEGFR2-VEGF complex to determine the Kd value (upper panel), the association and dissociation response curves of VEGFR2-VEGF assembly in the presence of Bevacizumab (0.1 μg/mL) or indicated concentrations of Aiphanol were generated (lower panel). (i) Western blot analysis of signaling proteins in HUVECs treated with 15 μM Aiphanol for indicated time. (j) Western blot analysis of signaling proteins in HUVECs treated with different amounts of Aiphanol for 1 h. Data represented mean ± SEM. * $P < 0.05$; ** $P < 0.01$; n.s., no significance.

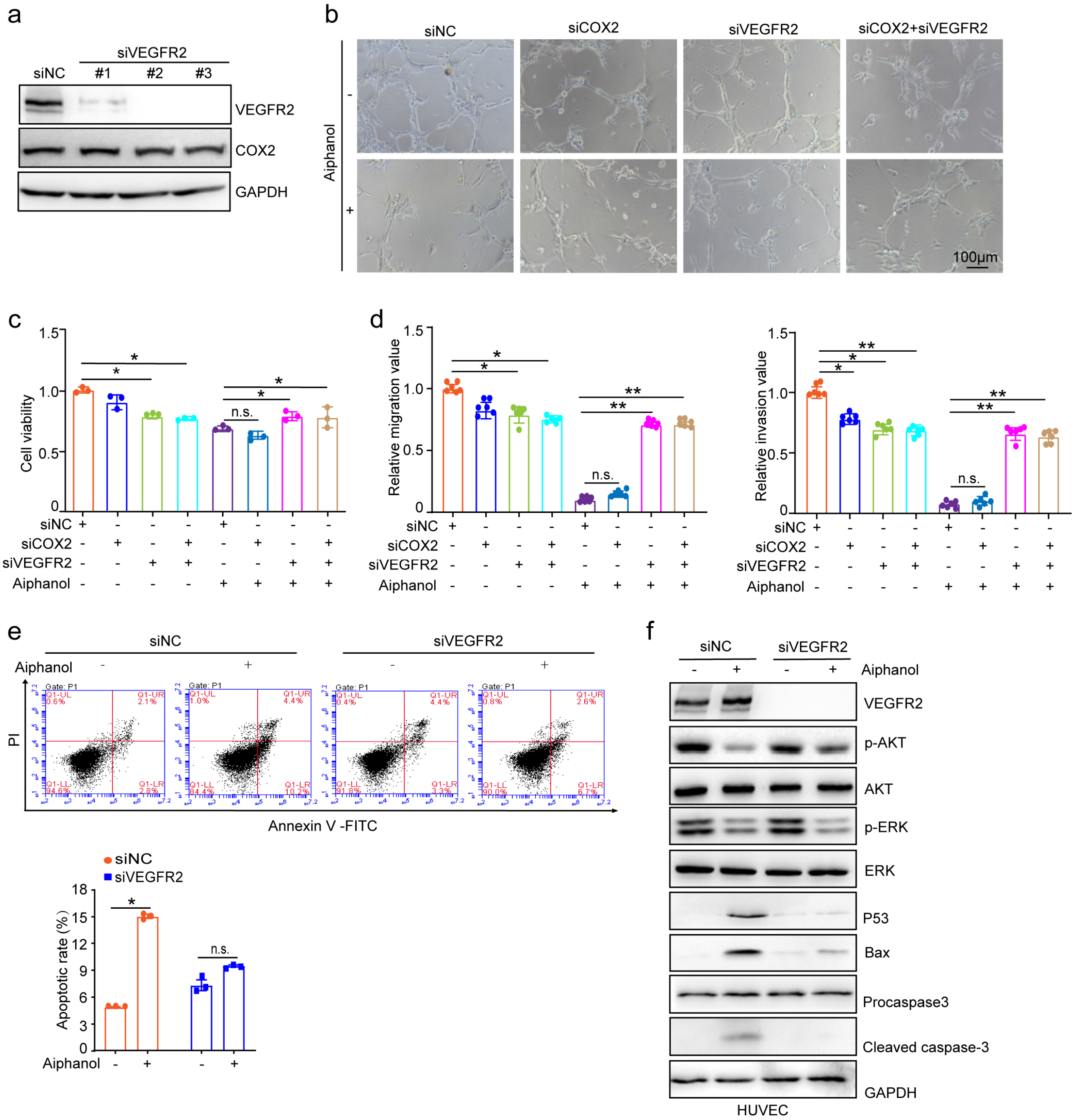

Supplementary Fig. S7. VEGFR2 and COX2 contribute to Aiphanol-inhibited angiogenesis *in vitro*.

(a) HUVECs were transfected with VEGFR2 siRNA or scrambled siRNA for 48 h, Western blot was used to detect the levels of VEGFR2 and COX2. (b) Representative photos showing the effects of VEGFR2 and COX2 silencing on Aiphanol-inhibited tube formation of HUVECs. (c) Effects of VEGFR2 and COX2 silencing on Aiphanol-inhibited viability of HUVECs. Cells were transfected with indicated siRNAs for 36 h, followed by Aiphanol (30 μM) treatment for 24 h (n = 3 per group). Cell viability was analyzed by CCK8 assay. (d) Effects of VEGFR2 and COX2 silencing on Aiphanol-inhibited migration and invasion of HUVECs. Cells were transfected with indicated siRNAs for 36 h, followed by transwell chamber assays for 24 h with or without

treatment of 30 μM Aiphanol (n = 6 per group). (e) Effects of VEGFR2 silencing on Aiphanol-induced apoptosis of HUVECs. Cells were transfected with indicated siRNAs for 48h, followed by Aiphanol (30 μM) treatment for 24 h (n = 3 per group). Apoptotic rates were analyzed by Annexin V/PI-staining. (f) Aiphanol-induced downregulation of AKT phosphorylation, upregulation of P53 and Bax, and cleavage of caspase3 were counteracted by VEGFR2 silencing in HUVECs. Cells were transfected with indicated siRNAs for 48h, followed by Aiphanol (30 μM) treatment for 24 h. Data represented mean ± SEM. *P < 0.05; **P < 0.01; n.s., no significance.

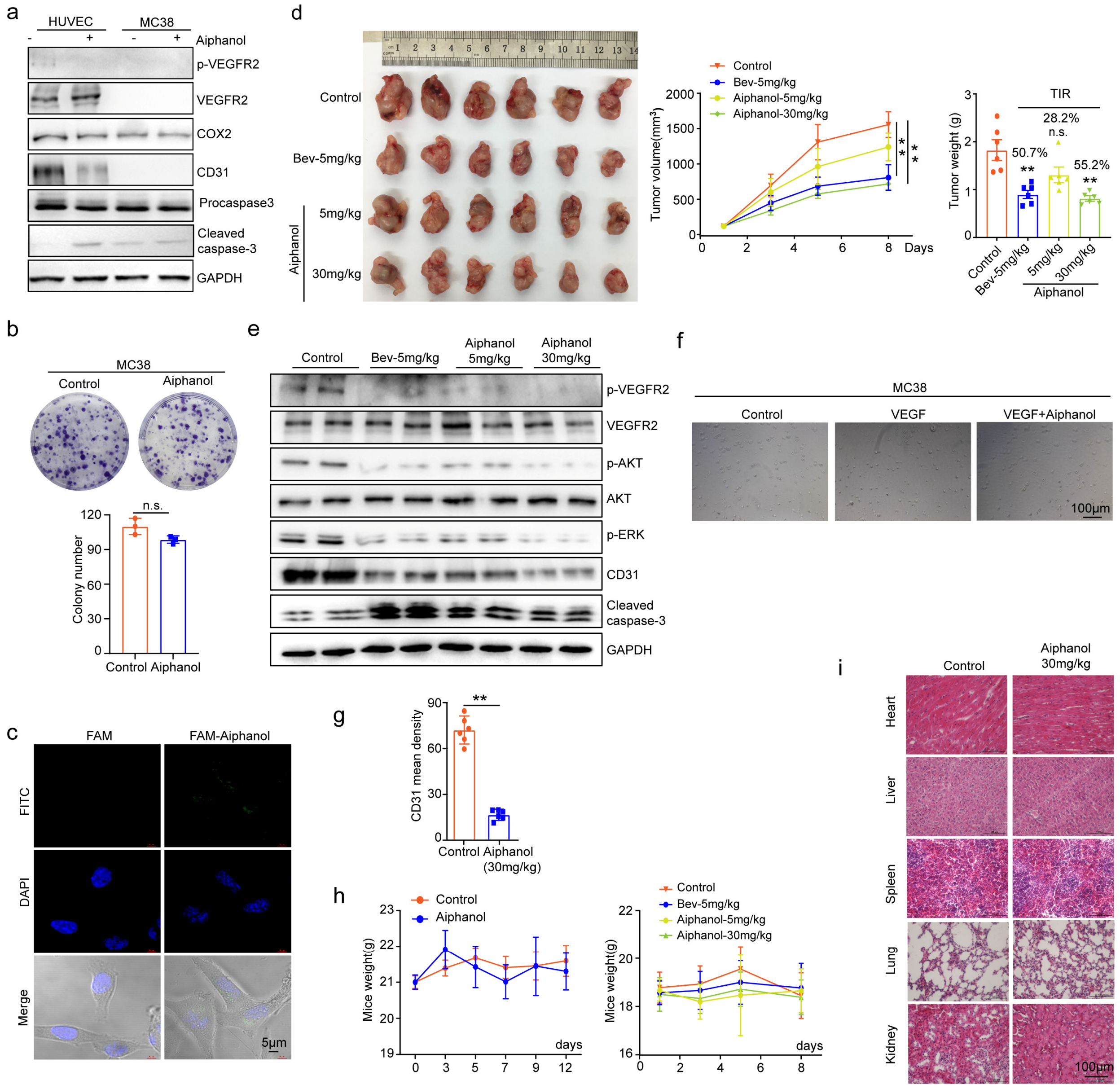

Supplementary Fig. S8. Aiphanol not only inhibits tumor growth in vivo but also has no harm to the hosts.

(a) Western blot analysis of indicated proteins in HUVECs and MC38 cells treated with 30 μM Aiphanol for 48 h. (b) Plate colony formation assay of MC38 cells treated with 30 μM Aiphanol for 12 d (n = 3 per group). (c) Uptake of FAM-Aiphanol (green) by MC38 cells. The nuclei were stained with Hoechst 33342 (blue). Scale bar, 5 μm. (d) Effect of double-dose (5 mg/kg and 30 mg/kg) treatment of Aiphanol on MC38 tumor growth in the syngeneic mouse model (n = 6 per group). Bevacizumab (Bev, 5 mg/kg) was used as the positive control. Left, macroscopic photo of dissected tumors; middle, growth curves of tumors; right, comparison of tumor weight at the endpoint. TIR, tumor inhibition rate. (e) Western blot analysis of indicated proteins in MC38 tumor tissues after double-dose treatment of Aiphanol (n = 2 per group). (f) Vasculogenic mimicry

analysis of MC38 cells to form VEGF-induced vascular channels *in vitro*. Scale bar, 100 μm. (g) Quantification of CD31 expression in the frozen sections of MC38 tumor tissues after single-dose (30 mg/kg) treatment of Aiphanol (n = 6 per group). (h) Changes in the body weight of Aiphanol-administrated C57BL/6 mice. Left panel, results of single-dose treatment; right panel, results of double-dose treatment. (i) Representative HE staining images of the heart, liver, spleen, lung and kidney from C57BL/6 mice after single-dose treatment of Aiphanol. Scale bar, 100 μm. Data represented the mean ± SEM. ** $P < 0.01$; n.s., no significance.

Supplementary Table S1.

Aiphanol's effects on the kinase activities in the *in vitro* Kinase profiler^{TM} assay

Kinase	Inhibition rate	Kinase	Inhibition rate	Kinase	Inhibition rate
ABL	62%	ERBB4	7%	MAPK1/ERK2	15%
ACK1	43%	FAK	7%	MAPK3/ERK1	34%
ACTR2	6%	FER	-1%	MAP4K3	34%
ALK	-14%	FES	-23%	MAP4K4	22%
ALK1	10%	FGFR1	51%	MAP4K5	19%
ARG	50%	FGFR2	27%	MEK1	0%
AMPKα1	7%	FGFR3	22%	MEK2	26%
AMPKα2	2%	FGFR4	7%	MARK1	14%
ASK1	8%	FGR	-7%	MARK3	22%
ATM	-9%	FLT1/VEGFR1	82%	MARK4	5%
ATR	26%	FLT3	76%	MEKK2	-9%
Aurora-A	17%	FLT4/VEGFR3	97%	MEKK3	9%
Aurora-B	67%	FMS	23%	MELK	28%
Aurora-C	2%	FYN	10%	MER	26%
AXL	45%	GSK3α	-5%	MET/HGFR	-47%
BLK	22%	GSK3β	15%	MKK3	22%
BMX	-23%	HCK	-7%	MKK6	4%
BRK	10%	HCK, activated	22%	MLCK	82%
BTK	26%	HIPK1	-5%	MLK1	70%
B-RAF	44%	HIPK2	6%	MLK2	35%
CaMKIV	25%	HIPK3	4%	MLK3	59%
CDC7/CyclinB1	1%	HPK1	54%	MLK4	-1%
CDK1/CyclinB	-4%	IGF1R	-22%	MNK2	26%
CDK2/CyclinA	-7%	IGF1R, activated	17%	MRCKα	16%
CDK5/p25	6%	IKKα	5%	MRCKβ	2%
CDK16/CyclinY	-26%	IKKβ	-4%	MRCKγ	5%
CHK1	15%	IKKε	4%	MSK1	7%
CHK2	48%	IR	-1%	MSK2	4%
CK2	-4%	IR, activated	18%	MST1	42%
CLK1	9%	IRR	22%	mTOR	-3%
cKit	34%	IRAK1	16%	mTOR/FKBP12	16%
C-RAF	43%	IRAK4	8%	NEK1	0%
DAPK1	1%	JAK1	10%	NEK2	8%
DDR1	27%	JAK2	4%	NEK4	51%
DDR2	6%	JAK3	5%	NEK3	29%
DYRK1A	26%	JNK1α1	-15%	NEK6	19%
DYRK1B	11%	JNK2α2	-10%	NEK7	4%
DYRK3	23%	JNK3	12%	NEK9	47%
EGFR	-2%	KDR/VEGFR2	85%	NEK11	83%
ERBB2/HER2	11%	LYN	9%	NLK	0%

Kinase	Inhibition rate	Kinase	Inhibition rate	Kinase	Inhibition rate
OSR1	-35%	PKCα	2%	ROS	-2%
p70S6K	54%	PKCβI	4%	RSK1	69%
PAK1	10%	PKCβII	19%	RSK2	60%
PAK2	21%	PKCγ	18%	RSK3	51%
PAK4	10%	PKCδ	-3%	RSK4	73%
PAK3	20%	PKCε	4%	SAPK2B	14%
PAK5	-5%	PKCη	-7%	SAPK3	-1%
PAK6	23%	PKCι	-11%	SAPK4	-14%
PDGFRα	15%	PKCμ	8%	SLK	10%
PDGFRβ	9%	PKCθ	-2%	SRPK2	6%
PDK1	18%	PKCζ	-6%	STK16	34%
PI3K(p110b/p85a)	10%	PKD2	17%	STK25	17%
PI3K(p120g)	-3%	PKD3	47%	STK39	-18%
PI3K(p110d/p85a)	19%	PKG1α	-1%	SYK	-6%
PI3K(p110a/p85a)	12%	PKG1β	16%	TAO1	0%
PI3K(p110a/p65a)	41%	PKR	17%	TAO2	21%
PI3KC2A	59%	PLK1	22%	TAO3	16%
PI3KC2G	21%	PLK4	18%	TBK1	31%
PIM3	7%	PRK1	49%	TEC, activated	45%
PIP4K2A	23%	PRK2	-2%	TGFBR1	2%
PIP5K1A	9%	PRKX	2%	TGFBR2	6%
PIP5K1G	8%	PYK2	12%	TIE2	6%
PKA	-22%	RET	80%	TNIK	12%
PKAcβ	-16%	RIPK2	56%	TRB2	-3%
PKBα	2%	ROCKI	9%	TTK	31%
PKBβ	-25%	ROCKII	34%	VRK1	-4%
PKBγ	17%	RON	37%	YES	30%

References

1. Gao Y, Su Y, Qu L, Xu S, Meng L, Cai SQ, Shou C. Mitochondrial apoptosis contributes to the anti-cancer effect of Smilax glabra Roxb. *Toxico.l Lett.* **207**, 112-120 (2011).
2. Feng J, Zhao C, Wang L, Qu L, Zhu H, Yang Z, An G, Tian H, Shou C. Feng J, Zhao C, Wang L, Qu L, Zhu H, Yang Z, An G, Tian H, Shou C. *Theranostics.* **8**, 2094-2106 (2018).
3. Lawson ND, Weinstein BM. In vivo imaging of embryonic vascular development using transgenic zebrafish. *Dev. Biol.* **248**, 307-318 (2002).
4. Prewett M, Huber J, Li Y, Santiago A, O'Connor W, King K, Overholser J, Hooper A, Pytowski B, Witte L, Bohlen P, Hicklin DJ. Antivascular endothelial growth factor receptor (fetal liver kinase 1) monoclonal antibody inhibits tumor angiogenesis and growth of several mouse and human tumors. *Cancer Res.* **59**, 5209-5218 (1999).
5. Baker M, Robinson SD, Lechertier T, Barber PR, Tavora B, D'Amico G, Jones DT, Vojnovic B, Hodivala-Dilke K. Use of the mouse aortic ring assay to study angiogenesis. *Nat. Protoc.* **7**, 89-104 (2011).
