# Supplementary figures and images for: Correction: Aiphanol, a native compound, suppresses angiogenesis via dual-targeting VEGFR2 and COX2
Source: Signal Transduct Target Ther. 2022 Apr 30;7:144. doi: 10.1038/s41392-022-00988-y (PMC9056504; doi:10.1038/s41392-022-00988-y)

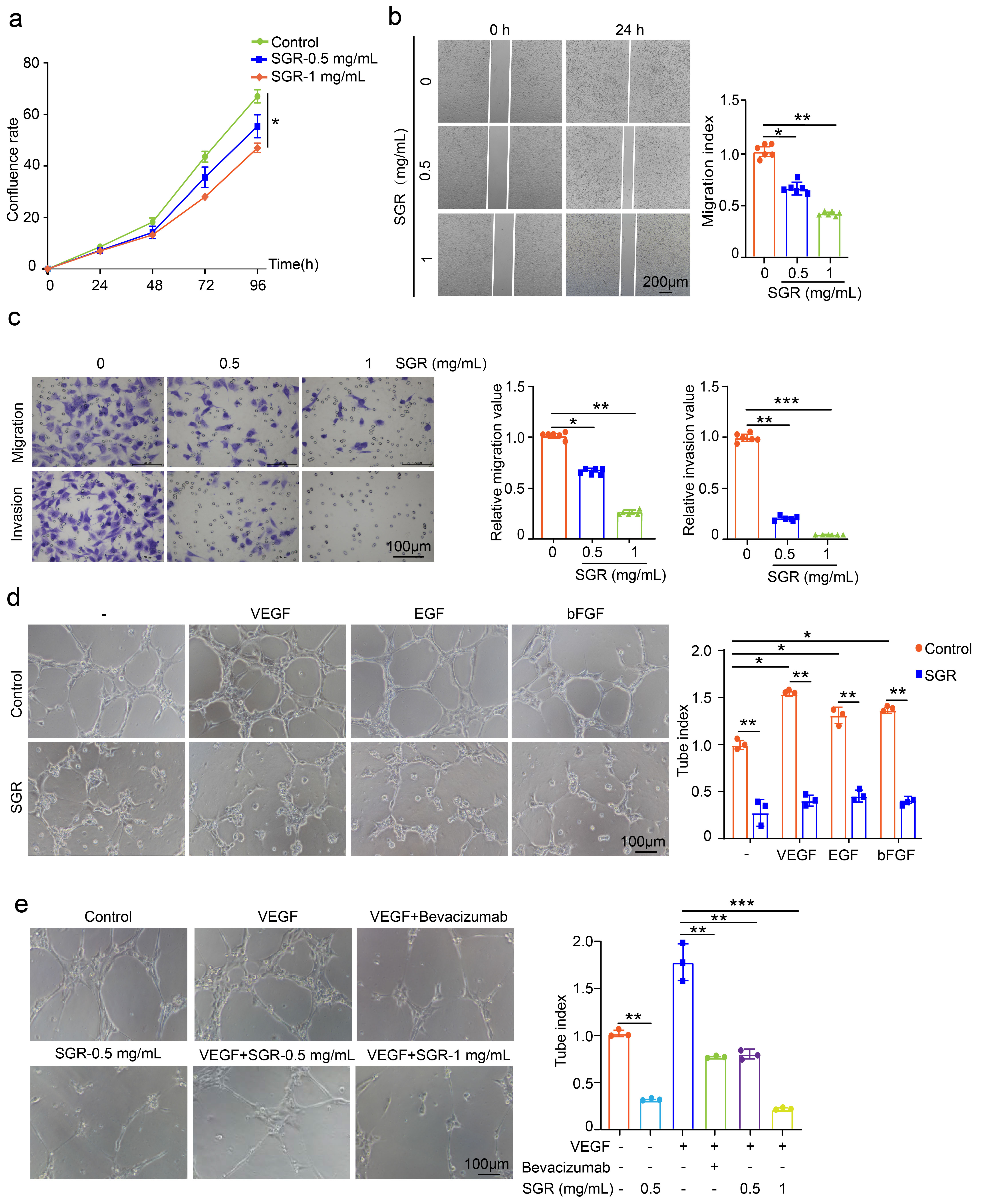

Supplement: Supplementary file 2 — Supplementary Fig S1c [file 41392_2022_988_MOESM2_ESM.jpg]

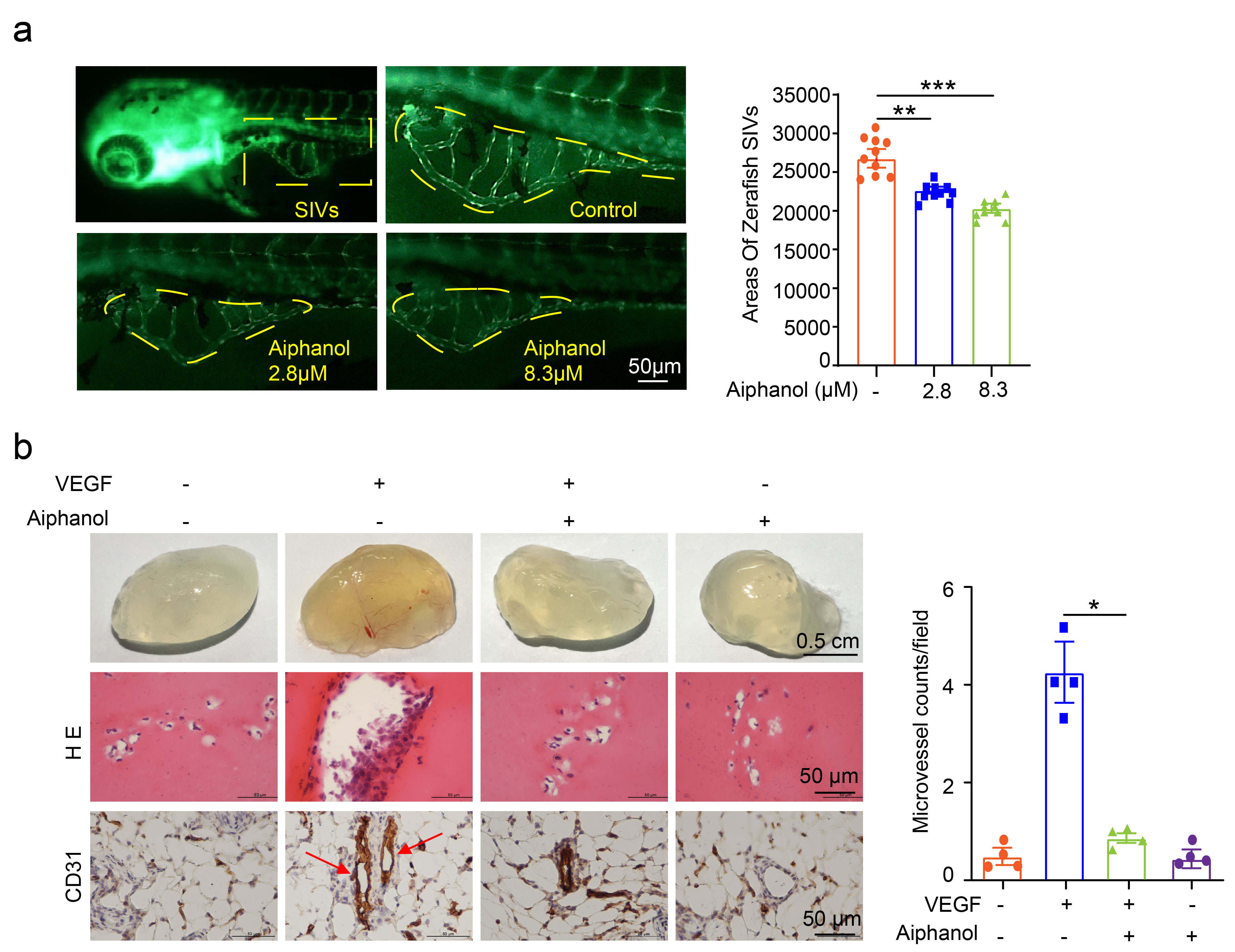

Supplement: Supplementary file 3 — Supplementary Fig S4b [file 41392_2022_988_MOESM3_ESM.jpg]
